# Combined PLT and NE to predict the prognosis of patients with locally advanced cervical cancer

**DOI:** 10.1038/s41598-020-66387-x

**Published:** 2020-07-08

**Authors:** Han Wang, Wen-Ming Chen, Yong-Heng Zhou, Jian-Ping Shi, Yue-qing Huang, Wen-Jie Wang

**Affiliations:** 1 Department of Oncology, Jining Cancer Hospital, Jining, Shandong 272000 P.R. China; 2 Department of Oncology, Jining No. 1 People’s Hospital; Affiliated Jining NO.1 People’s Hospital of Jining Medical University, Jining, Shandong 272011 P.R. China; 3 Department of Radio-Oncology, The Affiliated Suzhou Hospital of Nanjing Medical University, Suzhou, Jiangsu 215001 P.R. China; 4 Department of General Practice, The Affiliated Suzhou Hospital of Nanjing Medical University, Suzhou, Jiangsu 215001 P. R. China

**Keywords:** Cancer prevention, Gynaecological cancer

## Abstract

Cervical cancer is one of the most common tumors in women. Neutrophils (NEs) and platelets (PLTs) are components of cells in circulating blood. NEs are one of the components of white blood cells (WBCs), accounting for the vast majority of WBCs, recognized as one of the indicators of inflammation. PLTs are associated with thrombosis and inflammation. Both of them play an important role in tumor growth and metastasis. According to pre-radiotherapy PLT and NE media levels, we divided the patients into three groups: PLT and NE both high levels group, single high level group and both low group. By using COX regression models and nomogram, a prognostic model for patients was established. Both high levels of pre-radiotherapy PLT and NE group or high levels of post-radiotherapy PLT and NE group were correlated with worst overall survival (OS) compared with the other two groups. PLT and NE were correlated with outcomes of the patients with locally advanced cervical cancer.

## Introduction

In China, cervical cancer is one of the common tumors and the third most frequent cases of cancer mortality among female^1^. As one of the major cancer threaten to the health of female, the occurrence of cervical cancer is closely related to Human papillomavirus (HPV) infection^2^, of which high risk HPV16 and 18 are the most carcinogenic subtypes^3^. Surgery is widely accepted as the most efficient treatment for early cervical cancer, while locally advanced cervical cancer is mainly treated with concurrent chemoradiotherapy (CCRT)^4,5^. Although the prognosis of patients with cervical cancer has improved somewhat due to the improvement of surgery and radiotherapy techniques, a large proportion of patients with locally advanced stage still have recurrence and distant metastasis leading to treatment failure^6–8^. Therefore, effective, convenient, and inexpensive indicators are needed to predict the prognosis of these patients.

At present, the tumor microenvironment plays an important role in tumor growth and metastasis. A variety of inflammation-related cells, such as neutrophils, platelets, and lymphocytes, are important components of the tumor microenvironment^9^. Leukocytosis, especially the highest proportion of neutrophils in white blood cells (WBCs), is one of the paracancerous syndromes of many malignant tumors. Recent studies have indicated that neutrophils are one of the prognostic indicators in patients with recurrent or primarily treated cervical cancer^10,11^. It is well known that platelets play a significant role in the initiation and development of thrombosis and hemostasis^12^. High level of serum platelet levels has been previously found negatively correlated with be poor outcomes of cancers, including cervical, esophageal, renal, breast and lung cancer^13–16^.

Our research aims to combine serum PLT and NE levels to predict the prognosis of locally advanced cervical cancer patients, and hope to guide patients’ treatment through these indicators.

## Results

### Clinical features

120 patients were included in our study. All patients did not undergo surgery and chemotherapy prior to radiotherapy. The median age of the patients was 59.0 years and the median survival time was 41.5 months. Among them, 59/120 patients had lymph node metastasis. At the time of diagnosis, 20 patients were stage IB3, 43 patients were stage IIA2-IIB, and 57 patients were stage III. There were 29/120 patients with tumor diameter greater than 6 cm, and 91/120 with a diameter less than or equal to 6 cm. The clinicopathologic features was detailed in Tables 1 and 2. All patients underwent first-line CCRT.

**Table 1:** Clinicopathologic features.

Clinicopathologic features	n (120)	PLT			NE			
		Low (n)	High (n)	*P* value	Low (n)	High (n)	*P* value	
**Lymph nodes metastasis**								
none	61	42	20	0.845	34	28	0.056	
have	59	22	36		26	32		
**Age (years)**								
≤59	64	34	30	0.961	30	34	0.464	
>59	56	30	26		30	26		
**FIGO stage**								
IB3, IIA2, IIB	63	37	26	0.028*	37	26	0.043*	
IIIA, IIIB	57	22	35		23	34		
**Grade**								
1–2	86	41	45	0.092	46	40	0.224	
3	34	22	12		14	20		
**Tumor size**								
≤6 cm	91	54	37	0.019*	46	45	0.831	
>6 cm	29	10	19		14	15		

Abbreviations: FIGO, International Federation of Gynecology and Obstetrics; NE, neutrophils; PLT, platelets.

**Table 2:** Clinicopathologic features of PLT combination with NE.

Clinicopathologic features	n (120)	PLT combination with NE			
		Both low levels (n)	Single high level (n)	Both highs level (n)	*P* value
**Lymph nodes metastasis**					
none	61	29	18	15	0.002**
have	59	10	24	25	
**Age (years)**					
≤ 59	64	20	19	24	0.863
>59	56	22	20	15	
**FIGO stage**					
IB3, IIA2, IIB	63	25	25	13	0.027*
IIIA, IIIB	57	15	18	25	
**Grade**					
1–2	86	26	35	25	0.630
3	34	13	11	10	
**Tumor size**					
≤6 cm	91	33	34	24	0.015*
>6 cm	29	6	11	12	

Abbreviations: FIGO, International Federation of Gynecology and Obstetrics; NE, neutrophils; PLT, platelets.

**Figure 1:**
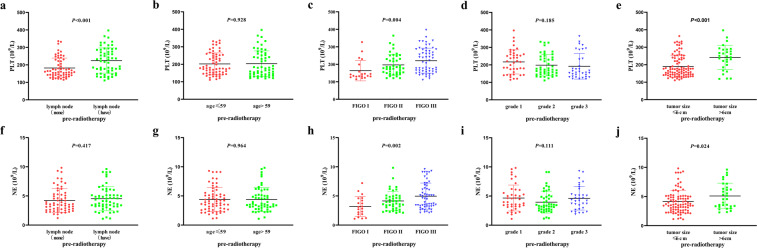
The relationship between pre-radiotherapy PLT and NE levels with different clinical features. (**a**) The pre-radiotherapy PLT levels and lymph node metastasis status. (**b**) The pre-radiotherapy PLT levels and ages. (**c**) The pre-radiotherapy PLT levels and FIGO stages. (**d**) The pre-radiotherapy PLT levels and grade. (**e**) The pre-radiotherapy PLT levels and tumor sizes. (**f**) The pre-radiotherapy NE levels and lymph node metastasis status. (**g**) The pre-radiotherapy NE levels and ages. (**h**) The pre-radiotherapy NE levels and FIGO stages. (**i**) The pre-radiotherapy NE levels and grade. (**j**) The p re-radiotherapy NE levels and tumor sizes.

We analyzed the relationship between clinical characteristics with PLT and NE show in Fig. 1. The pre-radiotherapy PLT levels had significantly differences in different lymph node metastasis, FIGO and tumor size groups. The pre-radiotherapy NE levels had significantly differences in different FIGO and tumor size groups.

### The survival analysis of locally advanced cervical cancer patients

**Figure 2:**
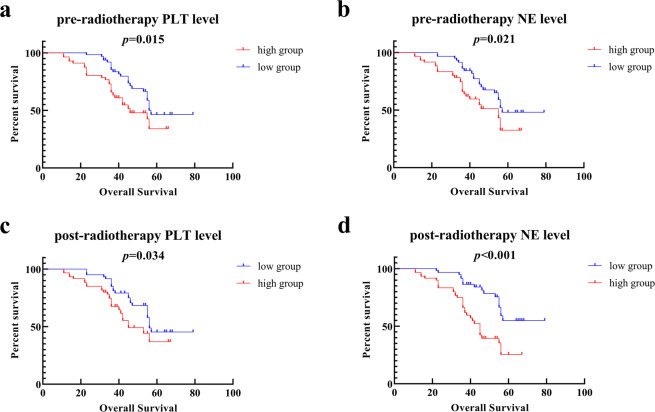
The relationships between PLT and NE with prognosis. (**a**) The OS of pre-radiotherapy high PLT level group compared to low level group. (**b**) The OS of pre-radiotherapy high NE level group compared to low level group. (**c**) The OS of post-radiotherapy high PLT level group compared to low level group. (**d**) The OS of post-radiotherapy high NE level group compared to low level group.

In pre-radiotherapy PLT and NE levels analysis, the patients were divided into two groups according to the median levels of PLT or NE. The high level PLT group had worth OS compared with low group show in Fig. 2a (*p* = 0.015, Hazard Ratio [HR] 1.902; 95% Confidence Interval [CI] 1.081–3.349). The high level NE group had worth OS compared to low level group show in Fig. 2b (*p* = 0.021, HR 1.855; 95% CI 1.070–3.216).In post-radiotherapy PLT and NE ratios analysis, the patients were divided into two groups according to the median levels of PLT or NE after CCRT. The high level of PLT group had worth OS compared to low level group show in Fig. 2c (*p* = 0.034, HR 1.749; 95% CI 1.002–3.060). The high level of NE group had worth OS compared to low level group show in Fig. 2d (*p* < 0.001, HR 2.811; 95% CI 1.615–4.895).

### The relationship between PLT and NE both high levels group or both increased ratios group with survival of cervical cancer patients

**Figure 3:**
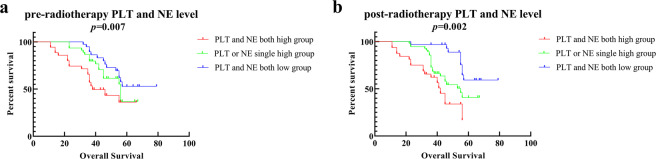
The relationships between different groups and prognosis. (**a**) The OS according to pre-radiotherapy different groups. (**b**) The OS according to post-radiotherapy different groups.

In pre-radiotherapy, the patients were divided into three groups (PLT and NE both high levels group, PLT or NE single high group, PLT and NE both low levels group). The both high levels of PLT and NE group had the worst OS compared with other two groups showed in Fig. 3a (*p* = 0.007). In the subgroup analysis, the both high levels group had worth OS compare to both low levels group show in Fig. S1a (*p* = 0.004).

In post-radiotherapy, the patients were divided into three groups (PLT and NE both high levels group, PLT or NE single high level group, PLT and NE both low level group). The both high levels of PLT and NE group had the worst OS compared to other two groups showed in Fig. 3b (*p* = 0.002). In the subgroup analysis, the both high levels group had worth OS compare to both low levels group show in Fig. S1d (*p* < 0.001), the single high levels group had worth OS compare to both low levels group show in Fig. S1f (*p* = 0.007).

### The COX analysis

**Figure 4:**
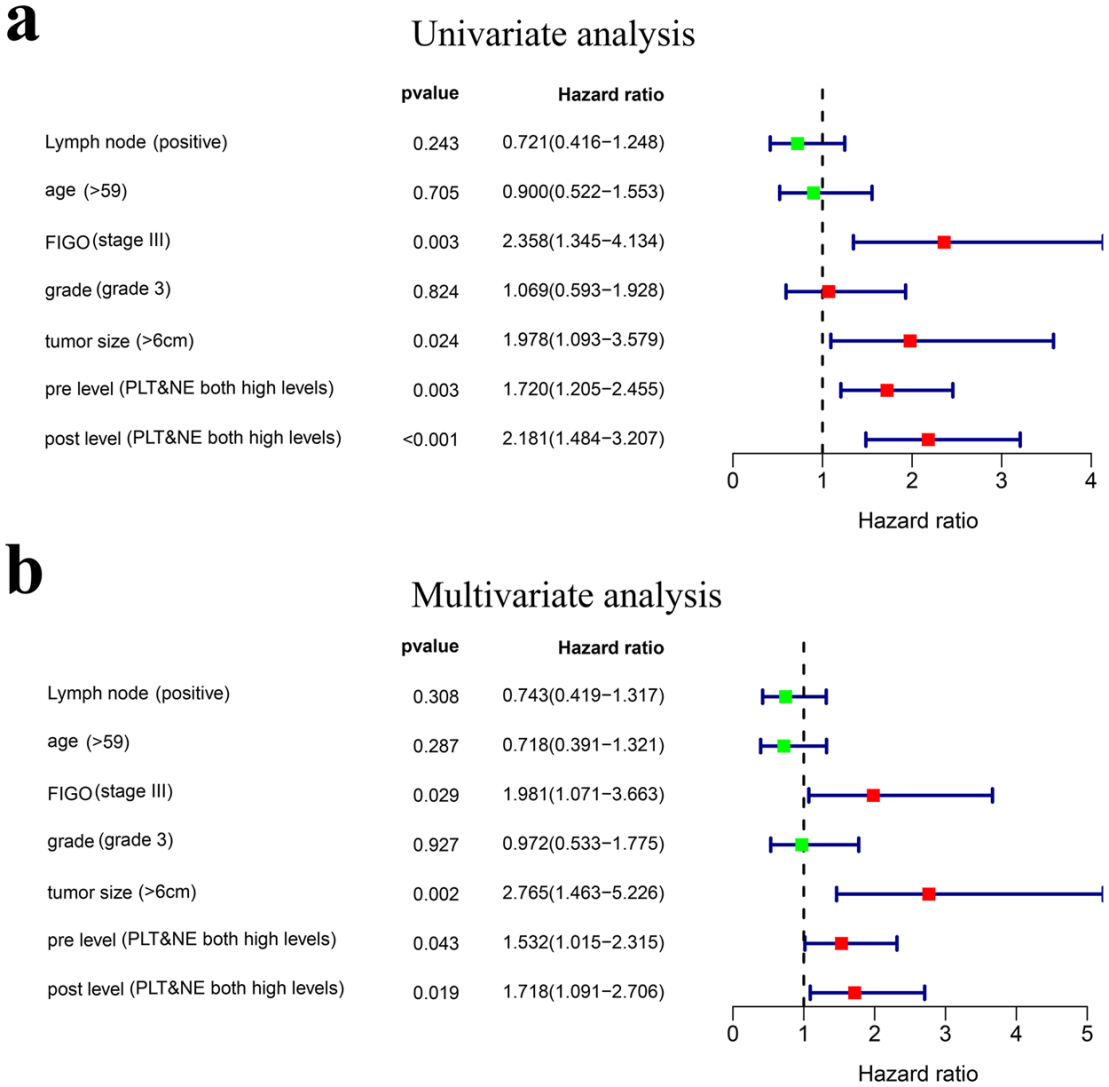
The COX analysis. (**a**) Univariate analyses. (**b**) Multivariate analysis.

In univariate COX analyses showed in Fig. 4a, the lymph node status (positive) (HR 0.721; 95% CI 0.416–1.248; *P* = 0.243), age (>59) (HR 0.900; 95% CI 0.522–1.553; *P* = 0.705) and grade status (HR 1.069; 95% CI 0.593–1.928; *P* = 0.824) were not risk factors for OS. The high FIOG stage (stage III) (HR 2.358; 95% CI 1.345–4.134; *P* = 0.003), tumor size (>6 cm) (HR 1.978; 95% CI 1.093–3.579; *P* = 0.024), pre-radiotherapy PLT and NE both high levels group (HR 1.720; 95% CI 1.205–2.455; *P* = 0.003), post-radiotherapy PLT and NE both high levels group (HR 2.181; 95% CI 1.484–3.207; *P* < 0.001) were significant risk factors for worth prognosis. In multivariate COX analysis showed in Fig. 4b, the high FIGO stage (stage III) (HR 1.981; 95% CI 1.071–3.663; *P* = 0.029), tumor size (>6 cm) (HR 2.765; 95% CI 1.463–5.226; *P* = 0.002), pre-radiotherapy PLT and NE both high levels group (HR 1.532; 95% CI 1.015–2.351; *P* = 0.043), post-radiotherapy PLT and NE both high levels group (HR 1.718; 95% CI 1.091–2.706; *P* = 0.019) were found to be independently associated with worth survival.

### The correlation of PLT with NE

**Figure 5:**
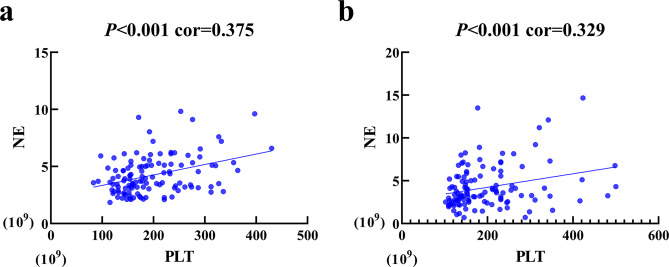
The relationships between PLT and NE. (**a**) Pre-radiotherapy levels of PLT and NE. (**b**) Post-radiotherapy levels of PLT and NE.

The pre-radiotherapy PLT level had positive correlation with NE level was showed in Fig. 5A (*p* < 0.001, cor=0.375). The post-radiotherapy PLT level had positive correlation with NE level was showed in Fig. 5B (*p* < 0.001, cor = 0.329).

### The prognostic Nomogram for cervical cancer patients

**Figure 6:**
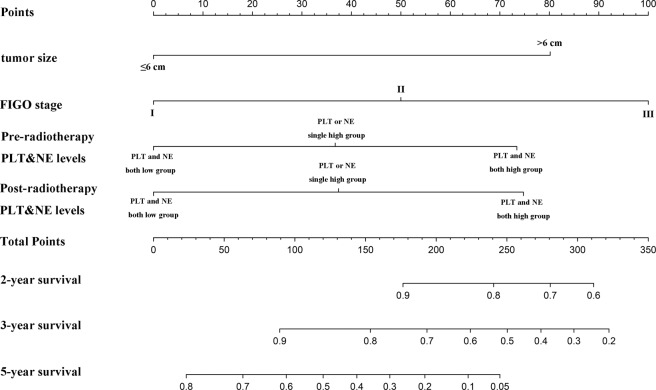
The Cervical cancer survival of nomogram. To use the nomogram, an individual patient’s value is located on each variable axis, and a line is drawn upward to determine the number of points received for each variable value. The sum of these numbers is located on the Total Points axis, and a line is drawn downward to the survival axes to determine the likelihood of 2-, 3- or 5-year survival.

Based on multivariate analysis, we integrated independent prognostic factors into the nomogram was showed in Fig. 6. The C-index was 0.712.

## Discussion

The present study integrated PLT and NE in outcome prediction and found that the combination of serum PLT and NE levels was independently correlated with prognosis of locally advanced cervical cancer patients after radical chemoradiotherapy. Escande *et al*. have demonstrated that, pre-treatment NE level (>7500**/**μl) was an independent risk factor for local failure-free survival in cervical cancer patients, suggesting that these patients have a poor prognosis^17^. Mabuchi *et al*. pointed out that, high pre-treatment level of NE(>8000**/**μl)in cervical cancer patients suggest a poor prognosis, and granulocyte colony stimulating factor (G-CSF) expression in tumor tissues is positively correlated with NE level^10^. Platelets are involved in multiple links of the process of thrombus formation and development. In fact, increased circulating PLT counts have been reported with unfavorable prognosis in pancreatic cancer^18–20^. Zheng *et al*. found that pre-operative PLT count could predict the prognosis of patients with early cervical cancer. In their study, patients were divided into four groups according to the cutoff pre-operative platelet value (PLT = 272 × 10^9^/L), combined with the FIGO stage. They found that patients with advanced FIGO stage (II) and a high PLT level (PLT ≥ 272 × 10^9^/L) had the worst prognosis^21^. Zhao *et al*.‘s study indicated that patients with high pre-operative levels of PLT and fibrinogen had a poor prognosis in early stage cervical cancer^22^. Cytokines secreted by tumors, such as Interleukin-1 (IL-1), IL-6, tumor necrosis factor-α (TNF-α), G-CSF and granulocyte-macrophage colony stimulating factor (GM-CSF) contribute to leukocytosis and neutrophilia^23,24^.These cytokines are closely related to inflammation and are now considered to be the key ingredients to tumor development^25^.

Several evidences support an important role for PLT in contributing to systematic inflammation and thrombosis promotion^26,27^. Serum PLT level is affected by both inherited and environmental factors^28–30^. It has been widely accepted that PLT is correlated with blood coagulation. A hypercoagulable state exists in cancers where elevated PLT counts can promote tumor development, angiogenesis and metastasis directly by secreting a series of proteins^26,31,32^. PLT can also facilitate cancer metastasis through cloaking disseminated cancer cells and preventing them from being cleared by immune system^33^. Therefore, more recently, increased attention has been given to investigate the relationship between PLT count and cancer. Both thrombocytosis and thrombocytopenia were identified to be a marker of poor prognosis in patients with cancer.

Elevated PLT or NE counts may be associated with chronic inflammation in patients with malignant tumor. The persistent infection of several viruses and bacteria are key risk factors for certain cancers, such as HPV infection can induce cervical cancer^3^ and Helicobacter pylori (HP) infection will increase the risk of developing gastric cancer^34^. Chronic inflammation can lead to local tissue damage, cell proliferation and transformation, but this does not necessarily lead to cancer^35^. These inflammatory cells can secrete TNF-α, high concentration of reactive oxygen species (ROS), macrophage migration inhibitory factor, and nitrogen^36^. Persistent can damage lead to DNA damage, further causing cell mutations and even transformation into tumor cells^36,37^.

A nomogram was performed to further verify our predictive model accuracy. Previous studies have found that nomogram might give a better outcome prediction in some tumors compared to traditional staging system^38,39^. In the present study, the C-index is 0.767, suggesting that the combined use of PLT and NE could be outcome indicator in advanced cervical cancer.

There are still some shortcomings in our research. Firstly, we all know that both chemotherapy and radiotherapy can cause bone marrow transplantation, especially concurrent radio-chemotherapy. The nutritional status of patients also affects hematopoietic function, which may have certain effects on post-treatment serum PLT and NE levels. In order to minimize the impact on the results, our post-treatment blood samples were collected tone month after radiotherapy. Secondly, our research is a retrospective study, which may have a certain shift in the results. Finally, the patients involved in the present study were from a single center. Multiple-center study will be performed to complete our research in further research.

In conclusion, our present study demonstrated that both high pre-radiotherapy PLT and NE levels and increased post/pre-radiotherapy PLT and NE ratios were correlated with worse OS in patients with advanced cervical cancer. By constructing a COX and nomogram model, we found that PLT and NE indicators can be used to predict the prognosis of patients with locally advanced cervical cancer.

## Methods

### Subjects

This retrospective study included a total of 120 patients with locally advanced cervical cancer who were admitted to the Jining Cancer Hospital from 2013 to 2015. All patients underwent systematic stage according to 2018 International Federation of Gynecology and Obstetrics (FIGO) stage system. OS was the main prognostic factor of this study. The study was approved by the Ethics Committee of Jining Cancer Hospital, and all patients signed an informed consent form. Our research is in compliance to the Declaration of Helsinki.

### Inclusion and exclusion criteria

The inclusion criteria: a) Patients with cervical cancer according to FIGO stage 1B3, IIB, IIIA and IIIB. b) Eastern Cooperative Oncology Group (ECOG) score: 0–1. c) Histopathology confirmed as squamous cell carcinoma. d) Age 18–75 years.

Exclusion criteria: a) Cervical Histopathology was non-squamous cell carcinoma. b) Patients failed to complete CCRT. c) Patients with fever, abnormally elevated CRP, have acute and chronic inflammation. d) Patient with acute and chronic bleeding or bleeding tendency, laboratory tests revealed abnormal blood coagulation.

### The detailed plan of CCRT of patients

Radiotherapy: In External Beam Radiation Therapy (EBRT), all patients were used Three-dimensional conformal radiation therapy (3D–CRT) or Intensity modulated radiation therapy (IMRT) technique. A total dose of 45.0–50.4 Gy in 25–28 fractions was prescribed. In intracavitary brachytherapy (ICBT), A cumulative dose of 30–36 Gy in 5–7 fractions was prescribed to point A.

Concurrent chemotherapy: The first line regimen of concurrent chemotherapy was cisplatin (30–40 mg/m^2^/week). Paclitaxel (60–80 mg/m^2^/week) was used for patients with who cannot tolerate cisplatin.

### The evaluation of patients

The patients was assessed by using Magnetic Resonance Imaging (MRI) and Computed Tomography (CT) scan every 3–6 months, and evaluated according to the criteria of Response Evaluation Criteria in Solid Tumors (RECIST) 1.1^40^.

### Blood samples

The patient was fasted for 8–10 hours, and then circulated blood to a sterile EDTA tube. By using a hematology analyser (Sysmex XE-2100; Sysmex, Kobe, Japan), patient’s blood samples were analyzed. In pre-radiotherapy analysis, the patients were divided into two groups according to the median value of PLT (low PLT, ≤ 183 × 10^9^/L or high PLT, > 183 × 10^9^/L), NE (low NE, ≤ 3.74 × 109/L or high NE, > 3.74 × 109/L). In post-radiotherapy analysis, the patients were divided into two groups according to the median value of PLT (low PLT, ≤ 169 × 10^9^/L or high PLT, > 169 × 10^9^/L), NE (low NE, ≤ 3.37 × 10^9^/L or high NE, > 3.37 × 10^9^/L).

### Follow-up

The survival time was calculated from the time of diagnosis until the patient dies or the last clinical follow-up. Patients were followed up for 8–76 months (media 38.5months).The first follow-up was one month after radiotherapy, until the patient dies or August 2019.

### Establishment of the nomogram

Establishment a nomogram based on multifactor analysis results by using edgeR package in the R environment (http://bioconductor.org/packages/edgeR/) (version3.53, R Development Core Team, Vienna, Austria). Use the C-index to evaluate the accuracy of the nomogram.

### Statistical analysis

Statistical analyses of the data were performed using SPSS (version 19.0, Chicago, USA) or GraphPad Prism (version 8.0, San Diego, USA). Independent prognostic factors were determined using a multivariate COX regression model. Patient survival time was analyzed using the Kaplan–Meier (KM) curve, and the log-rank test was used for statistical analysis. The association between PLT and NE was analyzed by Spearman’s rank correlation. Chi-Square test was used to compare the categorical variables. *P* < 0.05 was considered to have significant statistical difference.

## Supplementary information

Supplementary information.
