# Supplementary material for: Combined PLT and NE to predict the prognosis of patients with locally advanced cervical cancer
Source: Sci Rep. 2020 Jul 8;10:11210. doi: 10.1038/s41598-020-66387-x (PMC7343858; doi:10.1038/s41598-020-66387-x)
Supplement: Supplementary file 1 — Supplementary information. [file 41598_2020_66387_MOESM1_ESM.pdf]

Supplementary Information

Combined PLT and NE to predict the prognosis of patients with locally advanced cervical cancer

Han Wang[1&], Wen-Ming Chen[2&], Yong-Heng Zhou[1&], Jian-Ping Shi[3], Yue-qing Huang[4], Wen-Jie Wang[3*]

[1]Department of Oncology, Jining Cancer Hospital, Jining, Shandong, 272000, P.R. China
[2]Department of Oncology, Jining No. 1 People's Hospital; Affiliated Jining NO.1 People's Hospital of Jining Medical University, Jining, Shandong, 272011, P.R. China.
[3]Department of Radio-Oncology, The Affiliated Suzhou Hospital of Nanjing Medical University, Suzhou, Jiangsu, 215001, P.R. China
[4]Department of General Practice, The Affiliated Suzhou Hospital of Nanjing Medical University, Suzhou, Jiangsu 215001, P. R. China

[&]Authors contributed equally to this work

[*]Corresponding to:
Wen-Jie Wang M.D., E-mail: suda_wangwenjie@163.com, Department of Radio-Oncology, the Affiliated Suzhou Hospital of Nanjing Medical University, Suzhou, Jiangsu 215001, P.R. China

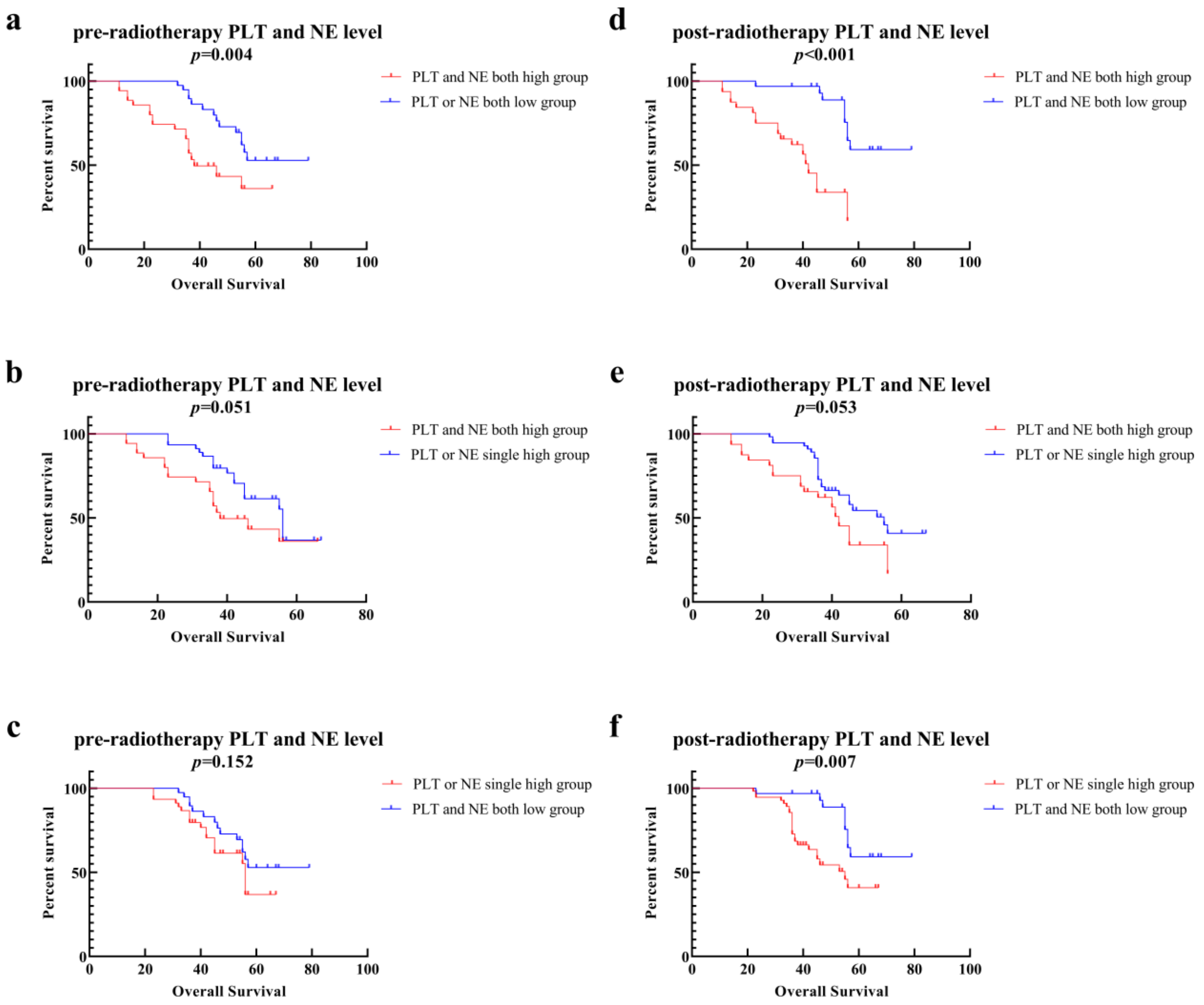

Supplementary Figure S1. The relationships between different groups of PLT and NE levels according to pre-radiotherapy and post-radiotherapy with overall survival (OS).

(a) The OS between pre-radiotherapy PLT and NE both high group with PLT and NE both low group.

(b) The OS between pre-radiotherapy PLT and NE both high group with PLT or NE single high group.

(c) The OS between pre-radiotherapy PLT and NE single high group with PLT and NE both low group.

(d) The OS between post-radiotherapy PLT and NE both high group with PLT and NE both low group.

(e) The OS between post-radiotherapy PLT and NE both high group with PLT or NE single high group.

(f) The OS between post-radiotherapy PLT and NE single high group with PLT and NE both low group.
